# Association of Age With Short-term and Long-term Mortality Among Patients Discharged From Intensive Care Units in France

**DOI:** 10.1001/jamanetworkopen.2019.3215

**Published:** 2019-05-10

**Authors:** Alice Atramont, Valérie Lindecker-Cournil, Jérémie Rudant, Ayden Tajahmady, Nicolas Drewniak, Annie Fouard, Mervyn Singer, Marc Leone, Matthieu Legrand

**Affiliations:** 1 Caisse Nationale d’Assurance Maladie (CNAM), Paris, France; 2 Bloomsbury Institute for Intensive Care Medicine, Division of Medicine, University College London, London, United Kingdom; 3 Aix Marseille Université, Assistance Publique Hôpitaux de Marseille, Service d’Anesthésie et de Réanimation, Hôpital Nord, Marseille, France; 4 Comité Réanimation de la Société Française d’Anesthésie et de Réanimation (SFAR), Paris, France; 5 L’Assistance Publique–Hôpitaux de Paris (AP-HP), Groupe Hospitalier St-Louis-Lariboisière, Department of Anesthesiology and Critical Care and Burn Unit, Paris, France; 6 University Paris Diderot, Paris, France; 7 Unité Mixte de Recherche Institut National de la Santé et de la Recherche Médicale (INSERM) 942, Investigation Network Initiative–Cardiovascular and Renal Clinical Trialists Network Paris, Paris, France

## Abstract

**Importance:**

An aging population is increasing the need for intensive care unit (ICU) beds. The benefit of ICU admission for elderly patients remains a subject of debate; however, long-term outcomes across all adult age strata are unknown.

**Objective:**

To describe short-term and long-term mortality (up to 3 years after discharge) across age strata in adult patients admitted to French ICUs.

**Design, Setting, and Participants:**

Using data extracted from the French national health system database, this cohort study determined in-hospital mortality and mortality at 3 months and 3 years after discharge of adult patients (older than 18 years) admitted to French ICUs from January 1 to December 31, 2013, focusing on age strata. The dates of analysis were November 2017 to December 2018.

**Exposure:**

Intensive care unit admission.

**Main Outcomes and Measures:**

In-hospital mortality and mortality at 3 months and 3 years after hospital discharge.

**Results:**

The study included 133 966 patients (median age, 65 years [interquartile range, 53-76 years); 59.9% male). Total in-hospital mortality was 19.0%, and 3-year mortality was 39.7%. For the 108 539 patients discharged alive from the hospital, 6.8% died by 3 months, and 25.8% died by 3 years after hospital discharge. After adjustment for sex, comorbidities, reason for hospitalization, and organ support (invasive ventilation, noninvasive ventilation, vasopressors, inotropes, fluid resuscitation, blood products administration, cardiopulmonary resuscitation, renal replacement therapy, and mechanical circulatory support), risk of mortality increased progressively across all age strata but with a sharp increase in those 80 years and older. In-hospital and 3-year postdischarge mortality rates, respectively, were 30.5% and 44.9% in patients 80 years and older compared with 16.5% and 22.5% in those younger than 80 years. Total 3-year mortality was 61.4% among patients 80 years and older vs 35.1% in those younger than 80. After age and sex standardization, excess mortality was highest among young patients during their first year after hospital discharge and persisted into the second and third years. In contrast, the mortality risk was close to the general population risk among elderly patients (≥80 years). Age and reason for hospitalization were strongly associated with long-term mortality (9-, 13-, and 20-fold increase in the risk of death 3 years after ICU discharge in patients aged 80-84, 85-89, and ≥90 years, respectively, compared with patients aged <35 years), while organ support use during ICU showed a weaker association (all organ support had 1.3-fold or lower increase in the risk of death).

**Conclusions and Relevance:**

Results of this study suggest that aging was associated with an increased risk of mortality in the 3 years after hospital discharge that included an ICU admission, with a sharp increase in those 80 years and older. However, compared with the general population matched by age and sex, excess long-term mortality was high in young surviving patients but not in elderly patients.

## Key Points

**Question:**

What is the association of age with long-term mortality after intensive care unit (ICU) discharge?

**Findings:**

In this cohort study that included 133 966 patients admitted to French ICUs, mortality at 3 years after discharge was 25.8%. Risk of mortality increased across all age strata after age 35 years but with a sharp increase in those 80 years and older; however, the mortality risk was close to the general population risk among elderly patients.

**Meaning:**

Aging was associated with an increased risk of mortality in the 3 years after hospital ICU discharge, while excess long-term mortality was highest in young surviving patients but not in elderly patients.

## Teaser

This cohort study using data extracted from the French national health system database describes short-term and long-term mortality (up to 3 years after discharge) across age strata in adult patients discharged from French intensive care units (ICUs).

## Introduction

Intensive care units (ICUs) are designed to care for patients with acute life-threatening conditions. An aging population, associated with an increasing incidence of patients with chronic comorbidities, increases the need for ICU beds in high-income countries.^1^ This represents a significant burden at both individual and collective levels. The benefit of ICU admission for elderly patients has been questioned^2,3,4^ because it may lead to unnecessary invasive care and avoidable health care expenditure.^5^ Consequently, there is an ongoing debate as to whether age should be used as a criterion for ICU admission.

In France, the number of ICU beds per 100 000 population is at the European mean,^6^ with no financial barriers to access ICU care because of a national health insurance system offering universal coverage for the French population. However, there are few wide-scale population-based studies that document short-term and long-term outcomes of adult patients after ICU discharge across all age strata. The use of a national claims database to document short-term and long-term outcomes of patients admitted to ICUs could provide essential information to the public, physicians, health care decision makers, and clinical researchers.

The main objective of our study was to describe the short-term and long-term mortality (in-hospital and at 3 months and 3 years after hospital discharge) in adult patients admitted to French ICUs in 2013. A specific focus was on the association of age.

## Methods

### Data Source

Data were obtained from the French national health system database (Système National des Données de Santé [SNDS], formerly named Système National d’Information Interrégimes de l’Assurance Maladie [SNIIRAM]). These sources comprise comprehensive, anonymized administrative health care data reimbursed by the French national health insurance system.^7^ The SNDS includes the French hospital discharge database (Programme de Médicalisation des Systèmes d’Information [PMSI]), containing inpatient diagnoses and procedures and linked to outpatient data (prescription drugs, health care professional visits, and laboratory or imaging investigations). The date of death is comprehensively reported for the 100% coverage scheme beneficiaries. The SNDS also includes data on patient status for the long-term diseases 100% coverage scheme. Hospital stays are classified by the Groupes Homogènes de Malades (GHM) system, a French adaptation of diagnosis related groups. Hospital and long-term disease diagnoses are coded according to the *International Statistical Classification of Diseases*, *10th Revision*. Procedures are coded according to the French common classification of medical procedures (Classification Commune des Actes Médicaux [CCAM]). This cohort study followed the Strengthening the Reporting of Observational Studies in Epidemiology (STROBE) reporting guideline (eFigure 1 in the Supplement). The use of SNDS data by the Caisse Nationale d’Assurance Maladie (CNAM) general health scheme fund has been approved by decree and by the French data protection authority (Commission Nationale de l’Informatique et des Libertés [CNIL]). Informed consent was waived. The CNAM has permanent access to SNDS data in application of the provisions of article R. 1461-12 of the French public health code. All data are deidentified.

### Study Population

All patients 18 years and older covered by the French national health insurance general scheme (ie, the main health insurance scheme covering about 76% of the population living in France) who were admitted to an ICU in a health care facility in France for at least 1 night between January 1 and December 31, 2013, were included in this observational study (eFigure 1 in the Supplement). Patients admitted to burn units and step-down units were not included. The dates of analysis were November 2017 to December 2018.

### Statistical Analysis

Characteristics of the population included age, sex, comorbidities, reason for hospitalization, ICU procedures, length of hospital stay, length of ICU stay, and Simplified Acute Physiology Score II. Data on comorbidities were derived from algorithms combining inpatient diagnoses, long-term disease information, and pharmacy reimbursement claims applied annually to each beneficiary of the general scheme. Comorbidities identified in 2012 were used in this study, referring to the year before the ICU stay in 2013. Detailed methods of the morbidity identification algorithms are publicly available in French.^8^ A summary presentation in English is also available.^9^

Crude mortality rates were calculated at the following prespecified end points: in-hospital, 3 months and 3 years after admission, and 3 months and 3 years after hospital discharge. The cohort was divided into 10 different age strata from 18 to 34 years to 90 years and older. Age- and sex-standardized mortality ratios (SMRs) were calculated for the first, second, and third years after hospital discharge for survivors of each period to enable comparison of the mortality rate between the study population and that of the French general population.^10^ The SMR is the ratio of observed deaths within the study population divided by expected deaths. Expected deaths were calculated from the mortality rate of the French general population applied to each stratum of age and sex. The 2014 mortality rate of the general population was used for the first year, the 2015 rate was used for the second year, and the 2016 rate was used for the third year.

Logistic regression models were used to assess factors associated with (1) in-hospital mortality, (2) mortality within 3 months of hospital discharge, and (3) mortality between 3 months and 3 years after hospital discharge. The first analysis was performed on the entire cohort. A second analysis was performed on hospital survivors, and a third analysis was conducted on 3-month survivors. Identical explanatory variables were included in all models, including demographics, comorbidities, reason for hospitalization, and ICU procedures. A mortality risk was calculated for each patient, and the population was then ranked into quintiles, from the lowest mortality probability (quintile 1) to the highest one (quintile 5). Population characteristics were described according to the quintiles of mortality risk for the 3 periods, as cited above. Logistic regression models were performed among patients younger than 80 years and those 80 years and older.^2^ Results are expressed as percentage or median and interquartile range (IQR), SMR and 95% CI, or odds ratio (OR) and 95% CI. Analyses were performed using SAS 9.2 software (associated with SAS Enterprise Guide version 7.13; SAS Institute Inc).

## Results

### Characteristics of Patients Admitted to ICUs

In 2013, a total of 208 275 patients were admitted to a French ICU, of whom 133 966 were included in the analysis (eFigure 1 in the Supplement). The median age was 65 years (IQR, 53-76 years), and 59.9% were male. Characteristics are listed in Table 1. Eighty-nine percent of patients were admitted from home, with 46.0% via the emergency department. Forty-eight percent were surgical patients, of whom 17.7% underwent cardiac surgery. Respiratory disease (16.3%) and cardiovascular disease (10.0%) were the 2 leading reasons for nonsurgical admissions. Reasons for hospital admission by age strata are shown in eFigure 2 in the Supplement.

**Table 1.** Characteristics of the Patients Included in the Study

Variable	Value
No.	133 966
Age category, y, No. (%)	
18-34	10 122 (7.6)
35-44	9514 (7.1)
45-54	17 120 (12.8)
55-64	27 899 (20.8)
65-69	16 144 (12.1)
70-74	14 130 (10.5)
75-79	15 754 (11.8)
80-84	13 820 (10.3)
85-89	7280 (5.4)
≥90	2183 (1.6)
Sex, No. (%)	
Male	80 296 (59.9)
Female	53 670 (40.1)
Comorbidities, No. (%)	
Heart failure	13 660 (10.2)
Cerebrovascular disease	6917 (5.2)
Diabetes	29 854 (22.3)
Active cancer	13 026 (9.7)
Dementia, including Alzheimer disease	3029 (2.3)
Chronic respiratory disease	26 915 (20.1)
End-stage renal disease	3378 (2.5)
Liver disease	7372 (5.5)
Surgical patients, No. (%)	63 704 (47.6)
Reason for hospitalization, No. (%)	
Cardiac surgery	23 712 (17.7)
Respiratory disease	21 879 (16.3)
Cardiovascular disease	13 444 (10.0)
Neurologic disease	7842 (5.9)
Noncardiac surgery	36 254 (27.1)
Poisoning	6317 (4.7)
Hepatogastroenterology	5306 (4.0)
Renal or metabolic disease	5377 (4.0)
Trauma and burn injuries	4387 (3.3)
Organ transplant	1832 (1.4)
Infectious disease	2930 (2.2)
Miscellaneous	4686 (3.5)
ICU procedures, No. (%)	
Invasive mechanical ventilation	81 303 (60.7)
Noninvasive mechanical ventilation	35 673 (26.6)
Vasopressors or inotropes	53 166 (39.7)
Fluid resuscitation	24 696 (18.4)
Administration of blood products	8948 (6.7)
Cardiopulmonary resuscitation with intubation	3277 (2.4)
Renal replacement therapy	14 918 (11.1)
Emergency external electrical cardioversion	1800 (1.3)
Intracranial pressure monitoring	2483 (1.9)
Mechanical circulatory support	1707 (1.3)
Length of hospital stay, median (IQR), d	13 (7-23)
Length of ICU stay, median (IQR), d	3 (2-8)
SAPS II, median (IQR)^a^	37 (26-52)
Missing data, No.^b^	454

Abbreviations: ICU, intensive care unit; IQR, interquartile range; SAPS II, Simplified Acute Physiology Score II.

^a^ The SAPS (Simplified Acute Physiology Score) II score ranges from 0 to 163 points, and higher scores indicates more severe disease.

^b^ The missing data apply only to SAPS II scores.

### Crude Mortality Rates and SMRs

**Figure 1.**
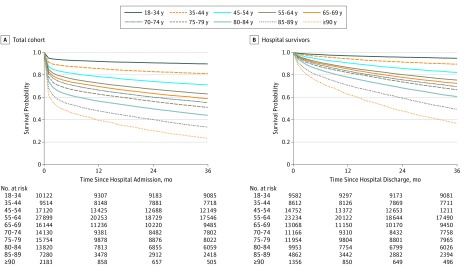
Kaplan-Meier Survival Curves of All Patients Since Hospital Admission and Hospital Discharge Survival is shown by age category.

Survival curves by age strata for the total cohort and for hospital survivors are shown in Figure 1A and B, respectively. The overall in-hospital mortality rate was 19.0%. The 3-month and 3-year mortality rates after hospital admission were 23.1% and 39.7%, respectively. The median survival of elderly patients (≥80 years) admitted to the ICU was 24 months for patients aged 80 to 84 years, 10 months for those aged 85 to 89 years, and 4 months for those 90 years and older. For the 108 539 patients discharged alive from the hospital, 6.8% died by 3 months, and 25.8% died by 3 years after hospital discharge. Among patients discharged alive, the median survival was 35 months for the cohort aged 85 to 89 years and 22 months for those 90 years and older. In-hospital and 3-year postdischarge mortality rates, respectively, were 30.5% and 44.9% in patients 80 years and older compared with 16.5% and 22.5% in those youger than 80 years. Total 3 years mortality was 61.4% among patients 80 years and older compared with 35.1% in patients younger than 80 years.

**Figure 2.**
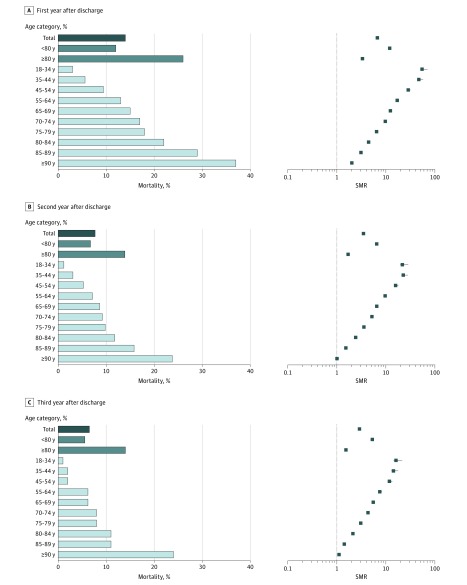
Mortality Rate and Standardized Mortality Ratio (SMR) in the First Year, Second Year, and Third Year After Hospital Discharge, by Age Category Error bars indicate 95% CIs.

After age and sex standardization, the SMRs were 6.64 (95% CI, 6.61-6.73) for the first year after hospital discharge, 3.50 (95% CI, 3.48-3.55) for the second year, and 2.86 (95% CI, 2.84-2.91) for the third year (Figure 2). Important variations of SMRs according to age strata were observed, with a higher SMR in the younger patients and a lower SMR in the older patients.

### Factors Associated With Mortality

#### In-Hospital Mortality

The multivariable analysis demonstrated that age, sex, most comorbidities, most reasons for hospitalization (using cardiac surgery as the reference), and most ICU procedures were associated with in-hospital mortality (Table 2). Female sex, a medical history of end-stage renal disease, and the use of noninvasive mechanical ventilation were associated with in-hospital survival.

**Table 2.** Factors Associated With Mortality in Logistic Regression Models

Variable	In-Hospital Vital Status (n = 133 966)			Vital Status Within 3 mo of Hospital Discharge for Hospital Survivors (n = 108 539)			Vital Status Between 3 mo and 3 y After Hospital Discharge for 3-mo Survivors (n = 101 207)		
	No. (%)		OR (95% CI)	No. (%)		OR (95% CI)	No. (%)		OR (95% CI)
	Alive (n = 108 539)	Dead (n = 25 427)		Alive (n = 101 207)	Dead (n = 7332)		Alive (n = 80 482)	Dead (n = 20 725)	
Age category, y									
18-34	9582 (8.8)	540 (2.1)	1 [Reference]	9452 (9.3)	130 (1.8)	1 [Reference]	9081 (11.3)	371 (1.8)	1 [Reference]
35-44	8612 (7.9)	902 (3.5)	1.80 (1.59-2.03)	8400 (8.3)	212 (2.9)	1.76 (1.41-2.20)	7711 (9.6)	689 (3.3)	1.98 (1.73-2.25)
45-54	14 752 (13.6)	2368 (9.3)	2.55 (2.28-2.84)	14 143 (14.0)	609 (8.3)	2.74 (2.25-3.32)	12 111 (15.0)	2032 (9.8)	3.31 (2.95-3.72)
55-64	23 234 (21.4)	4665 (18.3)	3.30 (2.97-3.66)	21 841 (21.6)	1393 (19.0)	3.88 (3.22-4.67)	17 490 (21.7)	4351 (21.0)	4.56 (4.08-5.10)
65-69	13 068 (12.0)	3076 (12.1)	4.05 (3.63-4.51)	12 168 (12.0)	900 (12.3)	4.48 (3.70-5.43)	9450 (11.7)	2718 (13.1)	5.19 (4.61-5.83)
70-74	11 166 (10.3)	2964 (11.7)	4.94 (4.43-5.51)	10 297 (10.2)	869 (11.9)	5.25 (4.33-6.36)	7758 (9.6)	2539 (12.3)	6.06 (5.38-6.82)
75-79	11 954 (11.0)	3800 (14.9)	6.18 (5.55-6.88)	10 874 (10.7)	1080 (14.7)	6.29 (5.20-7.61)	7965 (9.9)	2909 (14.0)	7.10 (6.31-7.98)
80-84	9953 (9.2)	3867 (15.2)	8.22 (7.38-9.16)	8838 (8.7)	1115 (15.2)	7.99 (6.61-9.66)	6026 (7.5)	2812 (13.6)	9.23 (8.20-10.39)
85-89	4862 (4.5)	2418 (9.5)	11.85 (10.57-13.29)	4106 (4.1)	756 (10.3)	10.85 (8.92-13.20)	2394 (3.0)	1712 (8.3)	13.41 (11.82-15.23)
≥90	1356 (1.2)	827 (3.3)	17.89 (15.54-20.58)	1088 (1.1)	268 (3.7)	14.10 (11.25-17.67)	496 (0.6)	592 (2.9)	20.70 (17.55-24.43)
Sex									
Male	64 851 (59.7)	15 445 (60.7)	1 [Reference]	60 373 (59.7)	4478 (61.1)	1 [Reference]	47 326 (58.8)	13 047 (63.0)	1 [Reference]
Female	43 688 (40.3)	9982 (39.3)	0.95 (0.91-0.98)	40 834 (40.3)	2854 (38.9)	0.87 (0.83-0.92)	33 156 (41.2)	7678 (37.0)	0.76 (0.73-0.78)
Comorbidities									
Heart failure	9770 (9.0)	3890 (15.3)	1.27 (1.21-1.34)	8479 (8.4)	1291 (17.6)	1.47 (1.37-1.58)	5004 (6.2)	3475 (16.8)	1.93 (1.83-2.04)
Cerebrovascular disease	5173 (4.8)	1744 (6.9)	1.10 (1.03-1.18)	4656 (4.6)	517 (7.1)	1.12 (1.02-1.24)	3222 (4.0)	1434 (6.9)	1.27 (1.18-1.36)
Diabetes	23 252 (21.4)	6602 (26.0)	0.97 (0.94-1.01)	21 252 (21.0)	2000 (27.3)	1.03 (0.97-1.09)	15404 (19.1)	5848 (28.2)	1.21 (1.16-1.26)
Active cancer	9729 (9.0)	3297 (13.0)	1.43 (1.36-1.51)	8391 (8.3)	1338 (18.2)	2.04 (1.90-2.18)	4761 (5.9)	3630 (17.5)	2.72 (2.59-2.86)
Dementia, including Alzheimer disease	1987 (1.8)	1042 (4.1)	1.36 (1.24-1.49)	1644 (1.6)	343 (4.7)	1.50 (1.33-1.71)	854 (1.1)	790 (3.8)	1.86 (1.67-2.07)
Chronic respiratory disease	20 821 (19.2)	6094 (24.0)	1.16 (1.11-1.21)	18 753 (18.5)	2068 (28.2)	1.14 (1.08-1.21)	12 520 (15.6)	6233 (30.1)	1.49 (1.43-1.55)
End-stage renal disease	2674 (2.5)	704 (2.8)	0.74 (0.67-0.83)	2407 (2.4)	267 (3.6)	1.24 (1.08-1.43)	1615 (2.0)	792 (3.8)	1.85 (1.67-2.05)
Liver disease	5304 (4.9)	2068 (8.1)	1.67 (1.57-1.79)	4750 (4.7)	554 (7.6)	1.62 (1.47-1.79)	3192 (4.0)	1558 (7.5)	2.02 (1.88-2.16)
Reason for hospitalization									
Cardiac surgery	22 339 (20.6)	1373 (5.4)	1 [Reference]	21 908 (21.6)	431 (5.9)	1 [Reference]	19 987 (24.8)	1921 (9.3)	1 [Reference]
Respiratory disease	16 688 (15.4)	5191 (20.4)	10.13 (9.39-10.93)	14 827 (14.7)	1861 (25.4)	6.67 (5.95-7.47)	10 060 (12.5)	4767 (23.0)	4.70 (4.40-5.02)
Cardiovascular disease	8969 (8.3)	4475 (17.6)	10.54 (9.76-11.38)	7986 (7.9)	983 (13.4)	5.45 (4.83-6.15)	5707 (7.1)	2279 (11.0)	3.70 (3.44-3.98)
Neurologic disease	5216 (4.8)	2626 (10.3)	19.57 (17.97-21.32)	4812 (4.8)	404 (5.5)	6.39 (5.53-7.38)	3947 (4.9)	865 (4.2)	3.57 (3.25-3.92)
Noncardiac surgery	30 068 (27.7)	6186 (24.3)	4.84 (4.50-5.20)	28 140 (27.8)	1928 (26.3)	3.75 (3.36-4.19)	21 328 (26.5)	6812 (32.9)	3.82 (3.60-4.05)
Poisoning	6056 (5.6)	261 (1.0)	2.31 (2.00-2.68)	5948 (5.9)	108 (1.5)	2.02 (1.62-2.51)	5371 (6.7)	577 (2.8)	2.61 (2.35-2.90)
Hepatogastroenterology	3878 (3.6)	1428 (5.6)	9.87 (8.95-10.89)	3375 (3.3)	503 (6.9)	7.98 (6.93-9.20)	2396 (3.0)	979 (4.7)	4.62 (4.20-5.09)
Renal or metabolic disease	4478 (4.1)	899 (3.5)	4.58 (4.11-5.11)	4022 (4.0)	456 (6.2)	5.56 (4.79-6.45)	2988 (3.7)	1034 (5.0)	3.77 (3.43-4.16)
Trauma and burn injuries	3561 (3.3)	826 (3.2)	8.86 (7.93-9.90)	3461 (3.4)	100 (1.4)	2.93 (2.33-3.68)	3256 (4.0)	205 (1.0)	1.53 (1.31-1.79)
Organ transplant	1677 (1.5)	155 (0.6)	1.15 (0.94-1.40)	1651 (1.6)	26 (0.4)	0.74 (0.49-1.11)	1508 (1.9)	143 (0.7)	0.79 (0.66-0.96)
Infectious disease	1920 (1.8)	1010 (4.0)	12.85 (11.47-14.40)	1691 (1.7)	229 (3.1)	7.01 (5.88-8.37)	1212 (1.5)	479 (2.3)	4.48 (3.95-5.09)
Miscellaneous	3689 (3.4)	997 (3.9)	14.19 (12.73-15.80)	3386 (3.3)	303 (4.1)	8.02 (6.83-9.42)	2722 (3.4)	664 (3.2)	5.34 (4.78-5.96)
ICU procedures									
Invasive mechanical ventilation	60 357 (55.6)	20 946 (82.4)	2.87 (2.75-2.99)	56 557 (55.9)	3800 (51.8)	1.08 (1.02-1.14)	46 253 (57.5)	10 304 (49.7)	1.04 (1.00-1.08)
Noninvasive mechanical ventilation	29 300 (27.0)	6373 (25.1)	0.71 (0.68-0.73)	26 716 (26.4)	2584 (35.2)	1.06 (1.01-1.12)	19 277 (23.9)	7439 (35.9)	1.22 (1.18-1.27)
Vasopressors or inotropes	34 865 (32.1)	18 301 (72.0)	2.88 (2.78-2.99)	31 785 (31.4)	3080 (42.0)	1.48 (1.40-1.57)	24 920 (31.0)	6865 (33.1)	1.10 (1.06-1.14)
Fluid resuscitation	17 520 (16.1)	7176 (28.2)	1.19 (1.15-1.24)	16 034 (15.8)	1486 (20.3)	1.11 (1.04-1.18)	12 462 (15.5)	3572 (17.2)	1.03 (0.98-1.08)
Administration of blood products	6039 (5.6)	2909 (11.4)	1.41 (1.33-1.50)	5576 (5.5)	463 (6.3)	1.22 (1.10-1.35)	4493 (5.6)	1083 (5.2)	1.11 (1.03-1.20)
Cardiopulmonary resuscitation with intubation	1125 (1.0)	2152 (8.5)	4.01 (3.68-4.38)	977 (1.0)	148 (2.0)	1.51 (1.25-1.82)	725 (0.9)	252 (1.2)	1.20 (1.02-1.40)
Renal replacement therapy	7606 (7.0)	7312 (28.8)	3.47 (3.32-3.64)	6650 (6.6)	956 (13.0)	1.48 (1.36-1.61)	4712 (5.9)	1938 (9.4)	1.17 (1.10-1.25)
Emergency external electrical cardioversion	866 (0.8)	934 (3.7)	1.39 (1.24-1.56)	770 (0.8)	96 (1.3)	1.12 (0.89-1.41)	590 (0.7)	180 (0.9)	0.96 (0.79-1.16)
Intracranial pressure monitoring	1794 (1.7)	689 (2.7)	2.06 (1.86-2.28)	1706 (1.7)	88 (1.2)	1.26 (1.01-1.58)	1549 (1.9)	157 (0.8)	0.69 (0.58-0.83)
Mechanical circulatory support	958 (0.9)	749 (2.9)	3.98 (3.51-4.52)	894 (0.9)	64 (0.9)	1.70 (1.29-2.23)	766 (1.0)	128 (0.6)	1.28 (1.05-1.57)

Abbreviations: ICU, intensive care unit; OR, odds ratio.

#### Long-term Mortality Among Patients Discharged Alive From the Hospital

**Figure 3.**
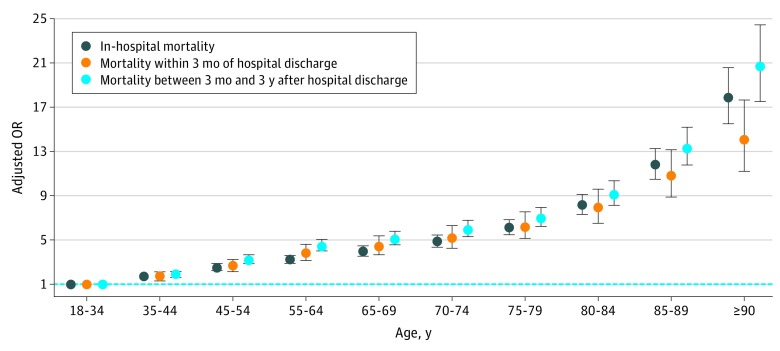
Association Between Age and Mortality After Adjustment for Sex, Comorbidities, Reason for Hospitalization, and Organ Support OR indicates odds ratio. Error bars represent 95% CIs, and the dashed line shows an OR of 1.

Factors in the multivariable analysis independently associated with 3-month and 3-year mortality after hospital discharge are listed in Table 2. Only a few marginal variables were not associated with an increased mortality. Risk of mortality increased across age strata, with a net inflection point above 80 years (Figure 3). Compared with cardiac surgery, the mortality risk was significantly higher for all reasons for hospitalization (except for organ transplant). Reasons for hospitalization had a lower association with long-term mortality (ie, 3 years after discharge alive) than with short-term mortality (ie, 3 months after discharge alive). Except for active cancer (OR, 2.04; 95% CI, 1.90-2.18 at 3 months and 2.72; 95% CI, 2.59-2.86 at 3 years), the 3-year mortality ORs of comorbidities were all below 2. Except for noninvasive mechanical ventilation, the association between organ support use and mortality was lower at 3 years than at 3 months after discharge alive. Associations between invasive mechanical ventilation or administration of blood products and mortality at 3 years after discharge were weak.

### Mortality in Patients 80 Years and Older

Patients 80 years and older accounted for 17.4% of the cohort (eTable 1 in the Supplement). Women (50.4%) were overrepresented in this age group compared with patients younger than 80 years. Comorbidities were more frequent in patients 80 years and older (except for liver disease). The median lengths of ICU and hospital stay, respectively, were 3 (IQR, 2-8) days and 12 (IQR, 7-23) days for patients younger than 80 years and 4 (IQR, 2-8) days and 14 (IQR, 7-23) days for elderly patients (≥80 years). With respect to ICU procedures, noninvasive mechanical ventilation and the use of vasopressors or inotropes were more frequent in the patients 80 years and older. In-hospital and 3-year postdischarge mortality rates, respectively, were 30.4% and 61.7% in the elderly patients compared with 16.5% and 35.3% in those younger than 80 years. Factors associated with mortality were similar regardless of age strata (eTable 2 and eTable 3 in the Supplement). Features of the population classified by their mortality risk are summarized in eTables 4, 5, and 6 in the Supplement. An analysis that excluded ICU procedures was performed as a sensitivity analysis. The association between age strata and outcome remained (eTable 7 in the Supplement).

## Discussion

Despite living in a high-income country with wide access to health care, approximately 40% of patients admitted to French ICUs did not survive 3 years. Even among hospital survivors, 6.8% died by 3 months, and 25.8% died by 3 years after hospital discharge. Our study shows an increase in short-term and long-term mortality risk after intensive care admission compared with an age-matched general population. A stepwise increase in mortality for all age strata was seen, with a sharp increase in risk for patients 80 years and older. Patients surviving until hospital discharge were 3 times more likely to die during the first 3 years than the general population after standardization by age and sex. However, excess mortality decreased with increasing age and tended to disappear in the oldest patients. Comorbidities and reasons for hospitalization were also associated with an increased long-term mortality. The use of organ support during ICU stay was not strongly associated with long-term mortality.

Our 3-year mortality rate after ICU discharge was close to the 21% reported by Hill et al^5^ from the Ontario, Canada, medical administrative database. Wunsch et al^11^ observed a 3-year mortality rate of 40% in a selected population of Medicare beneficiaries (ie, older than 65 years); this is intermediate to the 44.9% mortality we report in patients 80 years and older. Unsurprisingly, mortality was influenced by age.^12^ We found that in-hospital, 3-month, and 3-year mortality rates increased progressively with age from 18 years to 80 years, with a sharp increase after 80 years. This suggests that the threshold for defining elderly individuals with respect to ICU admission could be around 80 years. Conversely, the SMR also varied greatly across age strata but with an inverse association, being highest in young patients during the first year after hospital discharge. In contrast, the mortality risk was close to the general population risk after hospital discharge in the oldest patients.

At first sight, the observation of a higher mortality risk in older patients, yet lower in association with the general population matched for age, could appear contradictory. These results are likely associated with the higher life expectancy of younger people. After an acute event, the youngest patients have more years of lives lost in the event of death than elderly patients. A second factor is the role of senescence and underlying comorbidities facilitating the development of organ failure. Organ failures in young patients are expected to be mostly or entirely the result of the acute insult. In elderly individuals, and notwithstanding specific chronic organ disease, senescence (as a part of the natural aging process) will reduce functional reserve.^13,14^ As a result, a less severe acute illness may lead to organ failures due to a lack of organ functional reserve.^15,16,17^ The use of organ support (reflecting the severity of organ failure) was not associated with long-term survival in the elderly cohort herein. Therefore, the degree of senescence and impaired functional reserve may be more relevant in an elderly patient.^13^ The potential “gain” in survival after ICU admission is thus prominent.^2^ This point deserves particular consideration in the estimation of the potential benefit of interventions targeting critically ill ICU patients in clinical trials.^18^ Finally, a selection bias herein of elderly patients with perceived good functional status might explain, at least in part, why ICU survivors have a postdischarge outcome close to that of the general population not admitted to the ICU. Guidelines regarding ICU admission of elderly patients cannot be drawn from these data. However, this study provides important objective information on patient outcome for all age strata available for health care professionals, patients, and relatives.

The association of the use of organ support with outcome decreased after hospital discharge. Invasive mechanical ventilation and vasopressor or inotrope use were not associated with long-term mortality. These findings suggest that organ support use (except for renal replacement therapy) probably has little residual influence on long-term outcomes.^19^ Similarly, the ORs of comorbidities were low for in-hospital mortality but progressively increased at 3 months and at 3 years after discharge. This is in line with a prior study^20^ focusing on patients with septic shock in whom comorbidities were not the prominent determinant of short-term outcomes but were relevant later.

Finally, these results suggest that efforts are probably required to improve immediate management after ICU discharge, especially among younger patients. Such initiatives could include developing post–acute care facilities or appropriate home care in patients discharged from the ICU. Transition from acute care to home was likely underappreciated herein and may represent a factor for potential improvement in these patients. Other reasons, such as terminal illness or nonescalation of treatment, may also have a role in the early mortality seen after ICU discharge.

### Strengths and Limitations

A major strength of our study was the inclusion of a large number of patients using a nationwide database. Second, there was low loss to follow-up. Only those patients who were not French nationals and who died abroad were lost to follow-up with respect to survival. We chose to analyze postdischarge mortality in survivors separately from in-hospital mortality to avoid overrepresentation of factors associated with the hospital stay.

Our study has some limitations. First, its observational design prevents any causal association from being drawn. Second, only patients covered by the national health insurance general scheme were included. However, the general scheme covers about 76% of the French population. Some independent workers, farmers, students, and civil servants were not represented. Third, data on risk behavior (alcohol and tobacco) and body mass index were not available. We focused our analyses on the characteristics of patients and hospital stays at the time of initial ICU hospitalization, but events after hospital discharge and lifestyle could also alter long-term mortality risk. Factors likely to influence mortality, such as frailty, were not available. Such patients may have a greater degree of senescence. Fourth, selection bias probably influenced the decision to admit elderly patients to the ICU, to initiate ICU procedures, and to offer life-sustaining support. The decision to admit those elderly patients who were less frail likely selected those in whom active life-prolonging care was deemed appropriate. This selection bias could influence generalizability to all older patients. Immortal time bias may have altered in-hospital mortality with regard to offering ICU interventions. However, this bias is reduced because the patients had to spend at least 1 night in the ICU to be included, and most procedures were likely to be implemented early in their ICU stay. Nevertheless, we do not consider that immortal time influenced the long-term mortality analysis because all patients survived their ICU and hospital stay and had thus passed beyond the decision to have organ support offered. Also, we only assessed long-term mortality but not functional status or markers of quality of life.

## Conclusions

In this large national study performed in France, the overall survival of patients admitted to ICUs was approximately 60% at 3 years. Mortality rates increased progressively with age and more sharply in those 80 years and older. However, the SMR was especially high among the youngest patients immediately after ICU discharge, suggesting an area of improvement in this population and time frame. In contrast, elderly patients who survived to hospital discharge had a life expectancy much closer to that of the age-matched general population.

## Notes

Supplement. **eFigure 1.** Flow Chart of the Study Population Selection **eFigure 2.** Reasons for Hospitalization by Age **eTable 1.** Characteristics of the Patients by Age **eTable 2.** Factors Associated With Mortality in Logistic Regression Models Among Patients Aged Less Than 80 Years **eTable 3.** Factors Associated With Mortality in Logistic Regression Models Among Patients Aged 80 Years and Older **eTable 4.** Factors Associated With Mortality in Logistic Regression Models **eTable 5.** Characteristics of the Patients According to the 3-Month Mortality Risk After Hospital Discharge Among Hospital Survivors **eTable 6.** Characteristics of the Patients According to the 3-Year Mortality Risk After Hospital Discharge Among 3-Month Survivors **eTable 7.** Factors Associated With Mortality in Logistic Regression Models With ICU Procedures Not Considered
