# Supplementary material for: Association of Age With Short-term and Long-term Mortality Among Patients Discharged From Intensive Care Units in France
Source: JAMA Netw Open. 2019 May 10;2(5):e193215. doi: 10.1001/jamanetworkopen.2019.3215 (PMC6512465; doi:10.1001/jamanetworkopen.2019.3215)
Supplement: Supplement. — eFigure 1. Flow Chart of the Study Population Selection eFigure 2. Reasons for Hospitalization by Age eTable 1. Characteristics of the Patients by Age eTable 2. Factors Associated With Mortality in Logistic Regression Models Among Patients Aged Less Than 80 Years eTable 3. Factors Associated With Mortality in Logistic Regression Models Among Patients Aged 80 Years and Older eTable 4. Factors Associated With Mortality in Logistic Regression Models eTable 5. Characteristics of the Patients According to the 3-Month Mortality Risk After Hospital Discharge Among Hospital Survivors eTable 6. Characteristics of the Patients According to the 3-Year Mortality Risk After Hospital Discharge Among 3-Month Survivors eTable 7. Factors Associated With Mortality in Logistic Regression Models With ICU Procedures Not Considered [file jamanetwopen-2-e193215-s001.pdf]

Supplementary Online Content

Atramont A, Lindecker-Cournil V, Rudant J, et al. Association of age with short-term and long-term mortality among patients discharged from intensive care units in France. *JAMA Netw Open*. 2019;2(5):e193215. doi:10.1001/jamanetworkopen.2019.3215

eFigure 1. Flow Chart of the Study Population Selection

eFigure 2. Reasons for Hospitalization by Age

eTable 1. Characteristics of the Patients by Age

eTable 2. Factors Associated With Mortality in Logistic Regression Models Among Patients Aged Less Than 80 Years

eTable 3. Factors Associated With Mortality in Logistic Regression Models Among Patients Aged 80 Years and Older

eTable 4. Factors Associated With Mortality in Logistic Regression Models

eTable 5. Characteristics of the Patients According to the 3-Month Mortality Risk After Hospital Discharge Among Hospital Survivors

eTable 6. Characteristics of the Patients According to the 3-Year Mortality Risk After Hospital Discharge Among 3-Month Survivors

eTable 7. Factors Associated With Mortality in Logistic Regression Models With ICU Procedures Not Considered

This supplementary material has been provided by the authors to give readers additional information about their work.

© 2019 Atramont A et al. *JAMA Network Open*.

eFigure 1. Flow Chart of the Study Population Selection

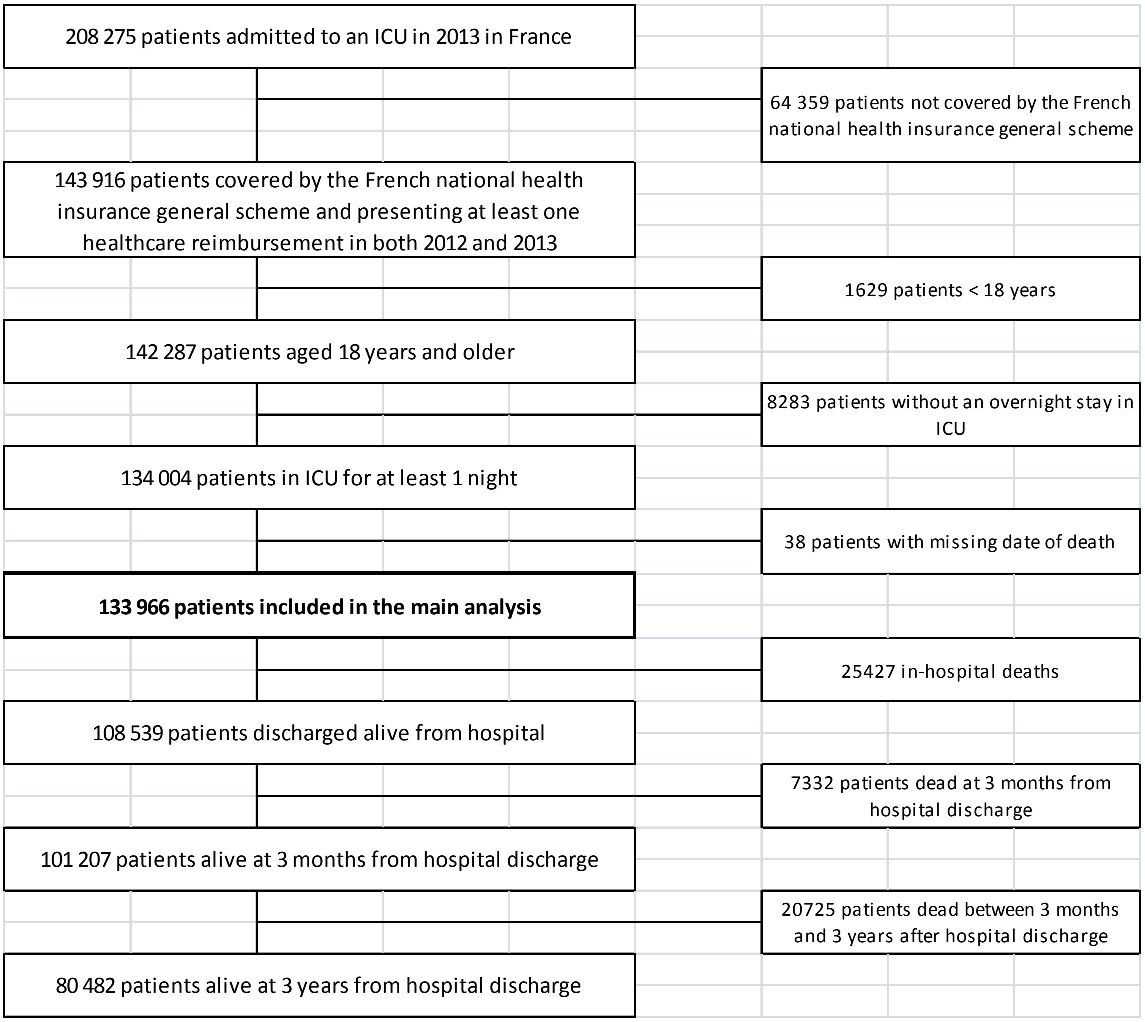

© 2019 Atramont A et al. *JAMA Network Open*.

eFigure 2. Reasons for Hospitalization by Age

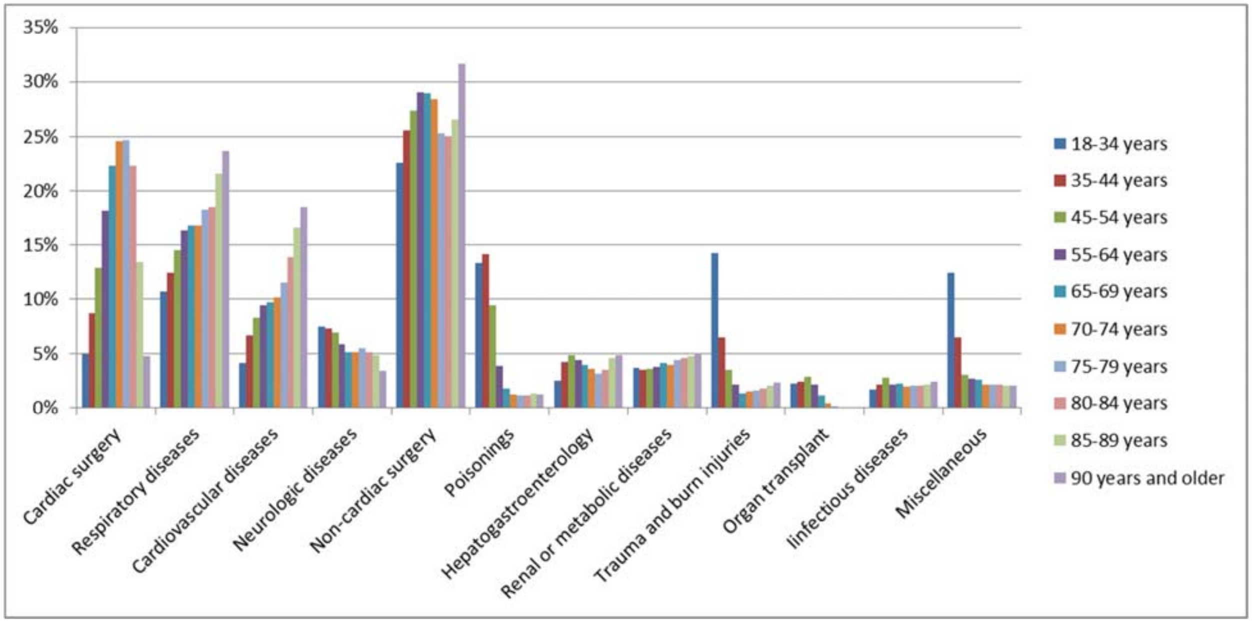

© 2019 Atramont A et al. *JAMA Network Open*.

eTable 1. Characteristics of the Patients by Age

	Less than 80 years			80 years and older	
	n = 110 683			n= 23 283	
	N	%		N	%
**Age (years)**					
18-34	10 122	**9.1**		.	**.**
35-44	9 514	**8.6**		.	**.**
45-54	17 120	**15.5**		.	**.**
55-64	27 899	**25.2**		.	**.**
65-69	16 144	**14.6**		.	**.**
70-74	14 130	**12.8**		.	**.**
75-79	15 754	**14.2**		.	**.**
80-84	.	**.**		13 820	**59.4**
85-89	.	**.**		7 280	**31.3**
90 and above	.	**.**		2 183	**9.4**
Median (IQR)	62 (50;70)			84 (81;86)	
**Sex**					
Male	68 738	**62.1**		11 558	**49.6**
Female	41 945	**37.9**		11 725	**50.4**
**Comorbidities**					
Heart failure	9 109	**8.2**		4 551	**19.5**
Cerebrovascular disease	5 244	**4.7**		1 673	**7.2**
Diabetes	23 807	**21.5**		6 047	**26.0**
Active cancer	10 752	**9.7**		2 274	**9.8**
Dementia (including Alzheimer's disease)	1 353	**1.2**		1 676	**7.2**
Chronic respiratory disease	21 198	**19.2**		5 717	**24.6**
End-stage renal disease	2 968	**2.7**		410	**1.8**
Liver diseases	7 012	**6.3**		360	**1.5**
**Reason for hospitalization**					
Cardiac surgery	19 551	**17.7**		4 161	**17.9**
Respiratory disease	17 240	**15.6**		4 639	**19.9**
Cardiovascular disease	9 915	**9.0**		3 529	**15.2**
Neurologic disease	6 710	**6.1**		1 132	**4.9**
Non-cardiac surgery	30 169	**27.3**		6 085	**26.1**
Poisoning	6 033	**5.5**		284	**1.2**
Hepatogastroenterology	4 374	**4.0**		932	**4.0**
Renal or metabolic disease	4 288	**3.9**		1 089	**4.7**
Trauma and burn injuries	3 938	**3.6**		449	**1.9**
Organ transplant	1 829	**1.7**		3	**0.0**
Infectious disease	2 433	**2.2**		497	**2.1**
Miscellaneous	4 203	**3.8**		483	**2.1**
**ICU procedures**					
Invasive mechanical ventilation	68 225	**61.6**		13 078	**56.2**
Non-invasive mechanical ventilation	27 859	**25.2**		7 814	**33.6**
Vasopressors or inotropes	43 240	**39.1**		9 926	**42.6**
Fluid resuscitation	20 675	**18.7**		4 021	**17.3**
Administration of blood products	7 595	**6.9**		1 353	**5.8**
Cardiopulmonary resuscitation with intubation	2 659	**2.4**		618	**2.7**
Renal replacement therapy	12 271	**11.1**		2 647	**11.4**
Emergency external electrical cardioversion	1 481	**1.3**		319	**1.4**
Intracranial pressure monitoring	2 417	**2.2**		66	**0.3**
Mechanical circulatory support	1 538	**1.4**		169	**0.7**
**Surgical patients**	53 533	**48.4**		10 171	**43.7**
**Length of hospital stay** - Median (IQR)	12 (7;23)			14 (7;23)	
**Length of ICU stay** - Median (IQR)	3 (2;8)			4 (2;8)	
**SAPS II** - Median (IQR)	35 (24;50)			45 (34;60)	
SAPS II- Missing data	378			76	

© 2019 Atramont A et al. *JAMA Network Open*.

eTable 2. Factors Associated With Mortality in Logistic Regression Models Among Patients Aged Less Than 80 Years

	In-hospital vital status						Vital status within 3 months after hospital discharge for hospital survivors						Vital status between 3 months and 3 years after hospital discharge for 3-month survivors					
	n = 110683						n = 92 368						n = 87 175					
	Alive		Dead				Alive		Dead				Alive		Dead			
	n = 92 368		n = 18 315				n = 87 175		n = 5 193				n = 71 566		n = 15 609			
	N	%	N	%	OR	95% CI	N	%	N	%	OR	95% CI	N	%	N	%	OR	95% CI
**Age (years)**																		
18-34	9 582	10.4	540	2.9	1.00	Ref.	9 452	10.8	130	2.5	1.00	Ref.	9 081	12.7	371	2.4	1.00	Ref.
35-44	8 612	9.3	902	4.9	1.75	[1.55-1.98]	8 400	9.6	212	4.1	1.73	[1.39-2.16]	7 711	10.8	689	4.4	1.96	[1.72-2.24]
45-54	14 752	16.0	2 368	12.9	2.46	[2.20-2.74]	14 143	16.2	609	11.7	2.67	[2.20-3.24]	12 111	16.9	2 032	13.0	3.27	[2.91-3.68]
55-64	23 234	25.2	4 665	25.5	3.18	[2.86-3.53]	21 841	25.1	1 393	26.8	3.77	[3.13-4.54]	17 490	24.4	4 351	27.9	4.51	[4.03-5.05]
65-69	13 068	14.1	3 076	16.8	3.90	[3.50-4.35]	12 168	14.0	900	17.3	4.35	[3.59-5.28]	9 450	13.2	2 718	17.4	5.14	[4.57-5.78]
70-74	11 166	12.1	2 964	16.2	4.79	[4.29-5.35]	10 297	11.8	869	16.7	5.10	[4.20-6.19]	7 758	10.8	2 539	16.3	6.02	[5.34-6.78]
75-79	11 954	12.9	3 800	21	6.02	[5.40-6.71]	10 874	12.5	1 080	21	6.1	[5.05-7.42]	7 965	11.1	2 909	18.6	7.09	[6.30-7.98]
**Sex**																		
Male	56 942	61.6	11 796	64.4	1.00	Ref.	53 578	61.5	3 364	64.8	1.00	Ref.	43 160	60.3	10 418	66.7	1.00	Ref.
Female	35 426	38.4	6 519	35.6	0.94	[0.90-0.97]	33 597	38.5	1 829	35.2	0.88	[0.83-0.93]	28 406	39.7	5 191	33.3	0.75	[0.72-0.78]
**Comorbidities**																		
Heart failure	6 816	7.4	2 293	12.5	1.29	[1.21-1.37]	6 077	7.0	739	14.2	1.50	[1.37-1.64]	3 906	5.5	2 171	13.9	1.91	[1.80-2.04]
Cerebrovascular disease	4 058	4.4	1 186	6.5	1.14	[1.05-1.23]	3 734	4.3	324	6.2	1.10	[0.97-1.24]	2 733	3.8	1 001	6.4	1.27	[1.17-1.38]
Diabetes	19 080	20.7	4 727	25.8	0.97	[0.93-1.02]	17 691	20.3	1 389	26.7	1.00	[0.94-1.08]	13 328	18.6	4 363	28.0	1.20	[1.15-1.26]
Active cancer	8 193	8.9	2 559	14.0	1.55	[1.46-1.64]	7 115	8.2	1 078	20.8	2.32	[2.15-2.50]	4 091	5.7	3 024	19.4	3.02	[2.86-3.19]
Dementia (including Alzheimer's disease)	951	1.0	402	2.2	1.36	[1.18-1.56]	821	0.9	130	2.5	1.60	[1.31-1.94]	494	0.7	327	2.1	1.87	[1.61-2.18]

© 2019 Atramont A et al. *JAMA Network Open*.

Chronic respiratory disease	16 946	**18.3**	4 252	**23.2**	**1.16**	[1.11-1.22]	15 513	**17.8**	1 433	**27.6**	**1.14**	[1.06-1.22]	10 865	**15.2**	4 648	**29.8**	**1.50**	[1.44-1.57]
End-stage renal disease	2 392	**2.6**	576	**3.1**	**0.77**	[0.69-0.87]	2 180	**2.5**	212	**4.1**	**1.27**	[1.08-1.49]	1 544	**2.2**	636	**4.1**	**1.79**	[1.60-2.00]
Liver disease	5 088	**5.5**	1 924	**10.5**	**1.67**	[1.55-1.78]	4 573	**5.2**	515	**9.9**	**1.60**	[1.44-1.77]	3 100	**4.3**	1 473	**9.4**	**2.04**	[1.89-2.19]
**Reason for hospitalization**																		
Cardiac surgery	18 522	**20.1**	1 029	**5.6**	**1.00**	Ref.	18 238	**20.9**	284	**5.5**	**1.00**	Ref.	16 906	**23.6**	1 332	**8.5**	**1.00**	Ref.
Respiratory disease	13 689	**14.8**	3 551	**19.4**	**9.80**	[8.96-10.71]	12 390	**14.2**	1 299	**25.0**	**7.45**	[6.49-8.56]	8 873	**12.4**	3 517	**22.5**	**5.03**	[4.67-5.43]
Cardiovascular disease	6 761	**7.3**	3 154	**17.2**	**11.36**	[10.38-12.44]	6 133	**7.0**	628	**12.1**	**6.21**	[5.36-7.20]	4 672	**6.5**	1 461	**9.4**	**3.91**	[3.59-4.27]
Neurologic disease	4 742	**5.1**	1 968	**10.7**	**18.12**	[16.42-19.98]	4 428	**5.1**	314	**6.0**	**6.79**	[5.73-8.04]	3 715	**5.2**	713	**4.6**	**3.72**	[3.35-4.13]
Non-cardiac surgery	25 813	**27.9**	4 356	**23.8**	**4.58**	[4.20-4.98]	24 462	**28.1**	1 351	**26.0**	**3.85**	[3.36-4.40]	19 098	**26.7**	5 364	**34.4**	**4.11**	[3.84-4.40]
Poisoning	5 812	**6.3**	221	**1.2**	**2.22**	[1.88-2.60]	5 723	**6.6**	89	**1.7**	**2.13**	[1.67-2.72]	5 213	**7.3**	510	**3.3**	**2.80**	[2.50-3.13]
Hepatogastroenterology	3 246	**3.5**	1 128	**6.2**	**10.82**	[9.67-12.12]	2 846	**3.3**	400	**7.7**	**9.92**	[8.40-11.71]	2 112	**3.0**	734	**4.7**	**4.99**	[4.47-5.57]
Renal or metabolic disease	3 689	**4.0**	599	**3.3**	**4.44**	[3.90-5.06]	3 386	**3.9**	303	**5.8**	**5.90**	[4.93-7.06]	2 644	**3.7**	742	**4.8**	**3.96**	[3.54-4.42]
Trauma and burn injuries	3 357	**3.6**	581	**3.2**	**7.06**	[6.21-8.03]	3 289	**3.8**	68	**1.3**	**2.60**	[1.97-3.42]	3 122	**4.4**	167	**1.1**	**1.60**	[1.35-1.90]
Organ transplant	1 674	**1.8**	155	**0.8**	**1.09**	[0.89-1.33]	1 648	**1.9**	26	**0.5**	**0.76**	[0.50-1.15]	1 505	**2.1**	143	**0.9**	**0.82**	[0.68-1.00]
Infectious disease	1 677	**1.8**	756	**4.1**	**12.80**	[11.24-14.57]	1 492	**1.7**	185	**3.6**	**8.05**	[6.57-9.85]	1 102	**1.5**	390	**2.5**	**4.89**	[4.26-5.62]
Miscellaneous	3 386	**3.7**	817	**4.5**	**14.60**	[12.93-16.49]	3 140	**3.6**	246	**4.7**	**8.85**	[7.35-10.67]	2 604	**3.6**	536	**3.4**	**5.54**	[4.90-6.26]
**ICU procedures**																		
Invasive mechanical ventilation	52 518	**56.9**	15 707	**85.8**	**3.12**	[2.96-3.29]	49 657	**57.0**	2 861	**55.1**	**1.08**	[1.01-1.15]	41 450	**57.9**	8 207	**52.6**	**1.07**	[1.02-1.12]
Non-invasive mechanical ventilation	23 752	**25.7**	4 107	**22.4**	**0.64**	[0.61-0.67]	22 021	**25.3**	1 731	**33.3**	**1.05**	[0.99-1.13]	16 648	**23.3**	5 373	**34.4**	**1.21**	[1.15-1.26]
Vasopressors or inotropes	29 575	**32.0**	13 665	**74.6**	**2.99**	[2.86-3.12]	27 265	**31.3**	2 310	**44.5**	**1.54**	[1.44-1.65]	21 924	**30.6**	5 341	**34.2**	**1.12**	[1.07-1.17]

© 2019 Atramont A et al. *JAMA Network Open*.

Fluid resuscitation	15 234	**16.5**	5 441	**29.7**	**1.19**	[1.14-1.24]	14 119	**16.2**	1 115	**21.5**	**1.09**	[1.01-1.17]	11 231	**15.7**	2 888	**18.5**	**1.07**	[1.02-1.12]
Administration of blood products	5 207	**5.6**	2 388	**13.0**	**1.48**	[1.38-1.58]	4 835	**5.5**	372	**7.2**	**1.33**	[1.18-1.50]	3 994	**5.6**	841	**5.4**	**1.11**	[1.02-1.21]
Cardiopulmonary resuscitation with intubation	975	**1.1**	1 684	**9.2**	**3.97**	[3.61-4.37]	860	**1.0**	115	**2.2**	**1.49**	[1.21-1.85]	655	**0.9**	205	**1.3**	**1.21**	[1.01-1.44]
Renal replacement therapy	6 550	**7.1**	5 721	**31.2**	**3.44**	[3.27-3.62]	5 806	**6.7**	744	**14.3**	**1.51**	[1.37-1.66]	4 287	**6.0**	1 519	**9.7**	**1.13**	[1.05-1.21]
Emergency external electrical cardioversion	730	**0.8**	751	**4.1**	**1.44**	[1.27-1.64]	656	**0.8**	74	**1.4**	**1.14**	[0.88-1.48]	519	**0.7**	137	**0.9**	**0.93**	[0.76-1.15]
Intracranial pressure monitoring	1 765	**1.9**	652	**3.6**	**2.11**	[1.90-2.35]	1 683	**1.9**	82	**1.6**	**1.31**	[1.03-1.65]	1 532	**2.1**	151	**1.0**	**0.68**	[0.57-0.81]
Mechanical circulatory support	872	**0.9**	666	**3.6**	**3.95**	[3.45-4.51]	817	**0.9**	55	**1.1**	**1.75**	[1.30-2.34]	706	**1.0**	111	**0.7**	**1.37**	[1.11-1.70]

© 2019 Atramont A et al. *JAMA Network Open*.

eTable 3. Factors Associated With Mortality in Logistic Regression Models Among Patients Aged 80 Years and Older

	**In-hospital vital status**						**Vital status within 3 months after hospital discharge for hospital survivors**						**Vital status between 3 months and 3 years after hospital discharge for 3-month survivors**					
	n = 23 283						n = 16 171						n = 14 032					
	**Alive**		**Dead**				**Alive**		**Dead**				**Alive**		**Dead**			
	n = 16171		n = 7112				n = 14 032		n = 2 139				n = 8 916		n = 5 116			
	N	**%**	N	**%**	**OR**	95% CI	N	**%**	N	**%**	**OR**	95% CI	N	**%**	N	**%**	**OR**	95% CI
**Age (years)**																		
80-84	9 953	**61.5**	3 867	**54.4**	**1.00**	Ref.	8 838	**63.0**	1 115	**52.1**			6 026	**67.6**	2 812	**55.0**		
85-89	4 862	**30.1**	2 418	**34.0**	**1.40**	[1.30-1.51]	4 106	**29.3**	756	**35.3**	**1.37**	[1.23-1.51]	2 394	**26.9**	1 712	**33.5**	**1.44**	[1.33-1.57]
90 and older	1 356	**8.4**	827	**11.6**	**2.03**	[1.82-2.27]	1 088	**7.8**	268	**12.5**	**1.76**	[1.51-2.06]	496	**5.6**	592	**11.6**	**2.24**	[1.95-2.56]
**Gender**																		
Male	7 909	**48.9**	3 649	**51.3**	**1.00**	Ref.	6 795	**48.4**	1 114	**52.1**	**1.00**	Ref.	4 166	**46.7**	2 629	**51.4**	**1.00**	Ref.
Female	8 262	**51.1**	3 463	**48.7**	**0.95**	[0.89-1.01]	7 237	**51.6**	1 025	**47.9**	**0.82**	[0.75-0.90]	4 750	**53.3**	2 487	**48.6**	**0.76**	[0.71-0.82]
**Comorbidities**																		
Heart failure	2 954	**18.3**	1 597	**22.5**	**1.26**	[1.17-1.37]	2 402	**17.1**	552	**25.8**	**1.46**	[1.31-1.64]	1 098	**12.3**	1 304	**25.5**	**1.98**	[1.80-2.18]
Cerebrovascular disease	1 115	**6.9**	558	**7.8**	**1.02**	[0.91-1.16]	922	**6.6**	193	**9.0**	**1.19**	[1.01-1.41]	489	**5.5**	433	**8.5**	**1.31**	[1.13-1.51]
Diabetes	4 172	**25.8**	1 875	**26.4**	**0.98**	[0.91-1.05]	3 561	**25.4**	611	**28.6**	**1.11**	[1.00-1.23]	2 076	**23.3**	1 485	**29.0**	**1.25**	[1.15-1.36]
Active cancer	1 536	**9.5**	738	**10.4**	**1.10**	[0.99-1.23]	1 276	**9.1**	260	**12.2**	**1.28**	[1.11-1.49]	670	**7.5**	606	**11.8**	**1.54**	[1.36-1.74]
Dementia (including Alzheimer's disease)	1 036	**6.4**	640	**9.0**	**1.33**	[1.18-1.50]	823	**5.9**	213	**10.0**	**1.42**	[1.21-1.68]	360	**4.0**	463	**9.1**	**1.82**	[1.56-2.12]
Chronic respiratory diseases	3 875	**24.0**	1 842	**25.9**	**1.13**	[1.04-1.22]	3 240	**23.1**	635	**29.7**	**1.13**	[1.01-1.26]	1 655	**18.6**	1 585	**31.0**	**1.44**	[1.32-1.58]
End-stage renal disease	282	**1.7**	128	**1.8**	**0.65**	[0.51-0.83]	227	**1.6**	55	**2.6**	**1.16**	[0.84-1.59]	71	**0.8**	156	**3.0**	**2.74**	[2.02-3.73]
Liver diseases	216	**1.3**	144	**2.0**	**1.40**	[1.09-1.80]	177	**1.3**	39	**1.8**	**1.29**	[0.90-1.85]	92	**1.0**	85	**1.7**	**1.39**	[1.01-1.91]
**Cause of hospitalization**																		

© 2019 Atramont A et al. *JAMA Network Open*.

Cardiac surgery	3 817	23.6	344	4.8	1.00	Ref.	3 670	26.2	147	6.9	1.00	Ref.	3 081	34.6	589	11.5	1.00	Ref.
Respiratory diseases	2 999	18.5	1 640	23.1	10.29	[8.89-11.90]	2 437	17.4	562	26.3	5.01	[4.08-6.14]	1 187	13.3	1 250	24.4	3.77	[3.29-4.31]
Cardiovascular diseases	2 208	13.7	1 321	18.6	8.35	[7.21-9.66]	1 853	13.2	355	16.6	4.03	[3.27-4.96]	1 035	11.6	818	16.0	3.03	[2.64-3.47]
Neurologic diseases	474	2.9	658	9.3	25.60	[21.40-30.61]	384	2.7	90	4.2	5.86	[4.40-7.82]	232	2.6	152	3.0	3.40	[2.70-4.29]
Non-cardiac surgery	4 255	26.3	1 830	25.7	5.49	[4.79-6.31]	3 678	26.2	577	27.0	3.53	[2.91-4.29]	2 230	25.0	1 448	28.3	2.98	[2.64-3.35]
Poisonings	244	1.5	40	0.6	2.71	[1.85-3.95]	225	1.6	19	0.9	2.08	[1.26-3.43]	158	1.8	67	1.3	2.07	[1.52-2.83]
Hepatogastroenterology	632	3.9	300	4.2	6.96	[5.70-8.51]	529	3.8	103	4.8	4.33	[3.28-5.73]	284	3.2	245	4.8	3.73	[3.04-4.58]
Renal or metabolic diseases	789	4.9	300	4.2	4.93	[4.03-6.03]	636	4.5	153	7.2	4.99	[3.83-6.50]	344	3.9	292	5.7	3.32	[2.71-4.06]
Trauma and burn injuries	204	1.3	245	3.4	20.58	[16.21-26.13]	172	1.2	32	1.5	4.71	[3.10-7.15]	134	1.5	38	0.7	1.42	[0.97-2.08]
Organ transplant	3	0.0	.	.	.	.	3	0.0	.	.	.	.	3	0.0	.	.	.	.
Iinfectious diseases	243	1.5	254	3.6	12.49	[9.85-15.85]	199	1.4	44	2.1	4.43	[3.04-6.46]	110	1.2	89	1.7	3.16	[2.32-4.30]
Miscellaneous	303	1.9	180	2.5	11.30	[8.86-14.41]	246	1.8	57	2.7	5.61	[3.99-7.89]	118	1.3	128	2.5	5.24	[3.98-6.92]
**ICU procedures**																		
Invasive mechanical ventilation	7 839	48.5	5 239	73.7	2.42	[2.25-2.62]	6 900	49.2	939	43.9	1.08	[0.97-1.21]	4 803	53.9	2 097	41.0	0.94	[0.86-1.02]
Non-invasive mechanical ventilation	5 548	34.3	2 266	31.9	0.92	[0.86-0.99]	4 695	33.5	853	39.9	1.10	[0.99-1.22]	2 629	29.5	2 066	40.4	1.27	[1.17-1.38]
Vasopressors or inotropes	5 290	32.7	4 636	65.2	2.63	[2.45-2.83]	4 520	32.2	770	36.0	1.30	[1.16-1.45]	2 996	33.6	1 524	29.8	1.03	[0.94-1.13]
Fluid resuscitation	2 286	14.1	1 735	24.4	1.22	[1.12-1.33]	1 915	13.6	371	17.3	1.15	[1.01-1.31]	1 231	13.8	684	13.4	0.86	[0.77-0.96]
Administration of blood products	832	5.1	521	7.3	1.17	[1.02-1.34]	741	5.3	91	4.3	0.90	[0.71-1.14]	499	5.6	242	4.7	1.08	[0.90-1.28]
Cardiopulmonary resuscitation with intubation	150	0.9	468	6.6	4.22	[3.42-5.20]	117	0.8	33	1.5	1.58	[1.04-2.38]	70	0.8	47	0.9	1.15	[0.77-1.71]
Renal replacement therapy	1 056	6.5	1 591	22.4	3.50	[3.16-3.88]	844	6.0	212	9.9	1.36	[1.14-1.63]	425	4.8	419	8.2	1.40	[1.19-1.64]

© 2019 Atramont A et al. *JAMA Network Open*.

Emergency external electric shock	136	**0.8**	183	**2.6**	**1.13**	[0.86-1.49]	114	**0.8**	22	**1.0**	**1.02**	[0.63-1.66]	71	**0.8**	43	**0.8**	**1.08**	[0.71-1.63]
Intracranial pressure monitoring	29	**0.2**	37	**0.5**	**2.46**	[1.46-4.14]	23	**0.2**	6	**0.3**	**1.85**	[0.74-4.60]	17	**0.2**	6	**0.1**	**0.71**	[0.27-1.86]
Mechanical circulatory support	86	**0.5**	83	**1.2**	**3.22**	[2.22-4.68]	77	**0.5**	9	**0.4**	**1.37**	[0.66-2.84]	60	**0.7**	17	**0.3**	**0.87**	[0.49-1.56]

© 2019 Atramont A et al. *JAMA Network Open*.

eTable 4. Factors Associated With Mortality in Logistic Regression Models

	Total		Quintiles									
			1		2		3		4		5	
	N	%	N	%	N	%	N	%	N	%	N	%
	133 966		27 036		26 552		26 792		26 808		26 778	
**Age (years)**												
18-34	10 122	**7.6**	5 856	**21.7**	1 944	**7.3**	1 439	**5.4**	720	**2.7**	163	**0.6**
35-44	9 514	**7.1**	3 600	**13.3**	2 318	**8.7**	1 664	**6.2**	1 406	**5.2**	526	**2.0**
45-54	17 120	**12.8**	4 508	**16.7**	4 322	**16.3**	3 023	**11.3**	3 330	**12.4**	1 937	**7.2**
55-64	27 899	**20.8**	5 706	**21.1**	6 459	**24.3**	5 396	**20.1**	5 872	**21.9**	4 466	**16.7**
65-69	16 144	**12.1**	2 617	**9.7**	3 884	**14.6**	2 986	**11.1**	3 301	**12.3**	3 356	**12.5**
70-74	14 130	**10.5**	2 152	**8.0**	2 817	**10.6**	2 990	**11.2**	2 821	**10.5**	3 350	**12.5**
75-79	15 754	**11.8**	1 841	**6.8**	2 318	**8.7**	4 026	**15.0**	3 178	**11.9**	4 391	**16.4**
80-84	13 820	**10.3**	582	**2.2**	1 988	**7.5**	3 470	**13.0**	3 157	**11.8**	4 623	**17.3**
85-89	7 280	**5.4**	165	**0.6**	472	**1.8**	1 545	**5.8**	2 113	**7.9**	2 985	**11.1**
90 and older	2 183	**1.6**	9	**0.0**	30	**0.1**	253	**0.9**	910	**3.4**	981	**3.7**
**Gender**												
Male	80 296	**59.9**	16 385	**60.6**	15 882	**59.8**	15 852	**59.2**	15 700	**58.6**	16 477	**61.5**
Female	53 670	**40.1**	10 651	**39.4**	10 670	**40.2**	10 940	**40.8**	11 108	**41.4**	10 301	**38.5**
**Comorbidities**												
Heart failure	13 660	**10.2**	909	**3.4**	1 841	**6.9**	2 898	**10.8**	3 365	**12.6**	4 647	**17.4**
Cerebrovascular disease	6 917	**5.2**	676	**2.5**	1 064	**4.0**	1 420	**5.3**	1 741	**6.5**	2 016	**7.5**
Diabetes	29 854	**22.3**	4 691	**17.4**	5 476	**20.6**	6 078	**22.7**	6 204	**23.1**	7 405	**27.7**
Active cancer	13 026	**9.7**	774	**2.9**	2 140	**8.1**	3 105	**11.6**	3 330	**12.4**	3 677	**13.7**
Dementia (including Alzheimer's disease)	3 029	**2.3**	59	**0.2**	194	**0.7**	500	**1.9**	974	**3.6**	1 302	**4.9**
Chronic respiratory diseases	26 915	**20.1**	2 496	**9.2**	5 456	**20.5**	6 138	**22.9**	6 073	**22.7**	6 752	**25.2**
End-stage renal disease	3 378	**2.5**	731	**2.7**	559	**2.1**	616	**2.3**	679	**2.5**	793	**3.0**
Liver diseases	7 372	**5.5**	418	**1.5**	1 100	**4.1**	1 589	**5.9**	1 813	**6.8**	2 452	**9.2**
**Cause of hospitalization**												
Cardiac surgery	23 712	**17.7**	12 327	**45.6**	6 302	**23.7**	3 230	**12.1**	1 212	**4.5**	641	**2.4**
Respiratory diseases	21 879	**16.3**	997	**3.7**	4 605	**17.3**	5 196	**19.4**	5 259	**19.6**	5 822	**21.7**
Cardiovascular diseases	13 444	**10.0**	178	**0.7**	1 479	**5.6**	2 488	**9.3**	3 746	**14.0**	5 553	**20.7**
Neurologic diseases	7 842	**5.9**	17	**0.1**	483	**1.8**	1 534	**5.7**	2 428	**9.1**	3 380	**12.6**
Non-cardiac surgery	36 254	**27.1**	6 102	**22.6**	7 830	**29.5**	8 401	**31.4**	8 252	**30.8**	5 669	**21.2**
Poisonings	6 317	**4.7**	3 864	**14.3**	1 445	**5.4**	706	**2.6**	241	**0.9**	61	**0.2**
Hepatogastroenterology	5 306	**4.0**	201	**0.7**	905	**3.4**	1 150	**4.3**	1 392	**5.2**	1 658	**6.2**

© 2019 Atramont A et al. *JAMA Network Open*.

Renal or metabolic diseases	5 377	**4.0**	1 084	**4.0**	1 134	**4.3**	1 257	**4.7**	1 058	**3.9**	844	**3.2**
Trauma and burn injuries	4 387	**3.3**	636	**2.4**	854	**3.2**	949	**3.5**	1 168	**4.4**	780	**2.9**
Organ transplant	1 832	**1.4**	823	**3.0**	377	**1.4**	318	**1.2**	213	**0.8**	101	**0.4**
Iinfectious diseases	2 930	**2.2**	86	**0.3**	324	**1.2**	518	**1.9**	751	**2.8**	1 251	**4.7**
Miscellaneous	4 686	**3.5**	721	**2.7**	814	**3.1**	1 045	**3.9**	1 088	**4.1**	1 018	**3.8**
**ICU procedures**												
Invasive mechanical ventilation	81 303	**60.7**	11 358	**42.0**	10 933	**41.2**	14 227	**53.1**	19 659	**73.3**	25 126	**93.8**
Non-invasive mechanical ventilation	35 673	**26.6**	5 990	**22.2**	7 873	**29.7**	7 904	**29.5**	7 625	**28.4**	6 281	**23.5**
Intravenous injection of dobutamine or dopamine	53 166	**39.7**	1 504	**5.6**	5 271	**19.9**	7 368	**27.5**	15 133	**56.4**	23 890	**89.2**
Intravenous infusion of filling product	24 696	**18.4**	2 391	**8.8**	3 262	**12.3**	4 136	**15.4**	6 099	**22.8**	8 808	**32.9**
Administration of blood products	8 948	**6.7**	527	**1.9**	1 052	**4.0**	1 607	**6.0**	2 183	**8.1**	3 579	**13.4**
Cardiopulmonary resuscitation with intubation	3 277	**2.4**	7	**0.0**	28	**0.1**	82	**0.3**	325	**1.2**	2 835	**10.6**
Renal replacement therapy	14 918	**11.1**	147	**0.5**	460	**1.7**	1 364	**5.1**	2 865	**10.7**	10 082	**37.7**
Emergency external electric shock	1 800	**1.3**	44	**0.2**	66	**0.2**	117	**0.4**	337	**1.3**	1 236	**4.6**
Intracranial pressure monitoring	2 483	**1.9**	25	**0.1**	97	**0.4**	479	**1.8**	1 206	**4.5**	676	**2.5**
Mechanical circulatory support	1 707	**1.3**	10	**0.0**	74	**0.3**	203	**0.8**	457	**1.7**	963	**3.6**
Q1: 2.84% ; Q2: 6.64% ; Q3:14.52% ; Q4: 34.54%												

© 2019 Atramont A et al. *JAMA Network Open*.

eTable 5. Characteristics of the Patients According to the 3-Month Mortality Risk After Hospital Discharge Among Hospital Survivors

	**Total**		**Quintiles**									
			**1**		**2**		**3**		**4**		**5**	
	N	**%**	N	**%**	N	**%**	N	**%**	N	**%**	N	**%**
	108 539		21 710		21 706		21 739		21 718		21 666	
**Age (years)**												
18-34	9 582	**8.8**	6 417	**29.6**	2 953	**13.6**	190	**0.9**	20	**0.1**	2	**0.0**
35-44	8 612	**7.9**	3 336	**15.4**	3 671	**16.9**	1 341	**6.2**	235	**1.1**	29	**0.1**
45-54	14 752	**13.6**	3 336	**15.4**	3 670	**16.9**	5 126	**23.6**	2 063	**9.5**	557	**2.6**
55-64	23 234	**21.4**	4 050	**18.7**	3 165	**14.6**	6 922	**31.8**	6 280	**28.9**	2 817	**13.0**
65-69	13 068	**12.0**	2 090	**9.6**	1 571	**7.2**	3 084	**14.2**	3 896	**17.9**	2 427	**11.2**
70-74	11 166	**10.3**	1 644	**7.6**	1 562	**7.2**	2 011	**9.3**	3 277	**15.1**	2 672	**12.3**
75-79	11 954	**11.0**	836	**3.9**	2 466	**11.4**	1 688	**7.8**	3 021	**13.9**	3 943	**18.2**
80-84	9 953	**9.2**	1	**0.0**	2 201	**10.1**	883	**4.1**	2 164	**10.0**	4 704	**21.7**
85-89	4 862	4	.	.	427	**2.0**	434	**2.0**	673	**3.1**	3 328	**15.4**
90 and older	1 356	1	.	.	20	**0.1**	60	**0.3**	89	**0.4**	1 187	**5.5**
**Gender**												
Male	64 851	**59.7**	13 354	**61.5**	12 164	**56.0**	12 742	**58.6**	13 086	**60.3**	13 505	**62.3**
Female	43 688	**40.3**	8 356	**38.5**	9 542	**44.0**	8 997	**41.4**	8 632	**39.7**	8 161	**37.7**
**Comorbidities**												
Heart failure	9 770	**9.0**	461	**2.1**	1 215	**5.6**	1 100	**5.1**	1 615	**7.4**	5 379	**24.8**
Cerebrovascular disease	5 173	**4.8**	404	**1.9**	707	**3.3**	924	**4.3**	1 276	**5.9**	1 862	**8.6**
Diabetes	23 252	**21.4**	3 028	**13.9**	3 596	**16.6**	4 039	**18.6**	5 684	**26.2**	6 905	**31.9**
Active cancer	9 729	**9.0**	70	**0.3**	691	**3.2**	1 248	**5.7**	2 456	**11.3**	5 264	**24.3**
Dementia (including Alzheimer's disease)	1 987	**1.8**	15	**0.1**	80	**0.4**	139	**0.6**	323	**1.5**	1 430	**6.6**
Chronic respiratory diseases	20 821	**19.2**	1 506	**6.9**	2 593	**11.9**	3 440	**15.8**	5 685	**26.2**	7 597	**35.1**
End-stage renal disease	2 674	**2.5**	500	**2.3**	303	**1.4**	361	**1.7**	519	**2.4**	991	**4.6**
Liver diseases	5 304	**4.9**	361	**1.7**	749	**3.5**	852	**3.9**	1 230	**5.7**	2 112	**9.7**
**Cause of hospitalization**												
Cardiac surgery	22 339	**20.6**	11 393	**52.5**	8 790	**40.5**	1 875	**8.6**	248	**1.1**	33	**0.2**
Respiratory diseases	16 688	**15.4**	471	**2.2**	1 186	**5.5**	2 385	**11.0**	5 610	**25.8**	7 036	**32.5**
Cardiovascular diseases	8 969	**8.3**	196	**0.9**	567	**2.6**	1 699	**7.8**	2 752	**12.7**	3 755	**17.3**
Neurologic diseases	5 216	**4.8**	349	**1.6**	787	**3.6**	1 399	**6.4**	1 551	**7.1**	1 130	**5.2**
Non-cardiac surgery	30 068	**27.7**	2 646	**12.2**	4 805	**22.1**	10 971	**50.5**	7 552	**34.8**	4 094	**18.9**

© 2019 Atramont A et al. *JAMA Network Open*.

Poisonings	6 056	**5.6**	3 669	**16.9**	1 773	**8.2**	462	**2.1**	132	**0.6**	20	**0.1**
Hepatogastroenterology	3 878	4	.	.	332	**1.5**	493	**2.3**	1 051	**4.8**	2 002	**9.2**
Renal or metabolic diseases	4 478	**4.1**	273	**1.3**	354	**1.6**	898	**4.1**	1 329	**6.1**	1 624	**7.5**
Trauma and burn injuries	3 561	**3.3**	1 594	**7.3**	1 015	**4.7**	633	**2.9**	253	**1.2**	66	**0.3**
Organ transplant	1 677	**1.5**	1 094	**5.0**	438	**2.0**	132	**0.6**	13	**0.1**	.	.
Iinfectious diseases	1 920	**1.8**	25	**0.1**	186	**0.9**	282	**1.3**	563	**2.6**	864	**4.0**
Miscellaneous	3 689	3	.	.	1 473	**6.8**	510	**2.3**	664	**3.1**	1 042	**4.8**
**ICU procedures**												
Invasive mechanical ventilation	60 357	**55.6**	14 029	**64.6**	13 883	**64.0**	10 924	**50.3**	10 465	**48.2**	11 056	**51.0**
Non-invasive mechanical ventilation	29 300	**27.0**	3 374	**15.5**	4 360	**20.1**	5 102	**23.5**	7 490	**34.5**	8 974	**41.4**
Intravenous injection of dobutamine or dopamine	34 865	**32.1**	3 798	**17.5**	7 295	**33.6**	5 665	**26.1**	7 254	**33.4**	10 853	**50.1**
Intravenous infusion of filling product	17 520	**16.1**	2 128	**9.8**	2 717	**12.5**	3 524	**16.2**	4 240	**19.5**	4 911	**22.7**
Administration of blood products	6 039	**5.6**	762	**3.5**	1 567	**7.2**	1 179	**5.4**	1 076	**5.0**	1 455	**6.7**
Cardiopulmonary resuscitation with intubation	1 125	**1.0**	39	**0.2**	130	**0.6**	154	**0.7**	218	**1.0**	584	**2.7**
Renal replacement therapy	7 606	**7.0**	280	**1.3**	742	**3.4**	1 007	**4.6**	1 770	**8.1**	3 807	**17.6**
Emergency external electric shock	866	**0.8**	57	**0.3**	128	**0.6**	153	**0.7**	170	**0.8**	358	**1.7**
Intracranial pressure monitoring	1 794	**1.7**	278	**1.3**	499	**2.3**	565	**2.6**	349	**1.6**	103	**0.5**
Mechanical circulatory support	958	**0.9**	110	**0.5**	293	**1.3**	257	**1.2**	123	**0.6**	175	**0.8**

Q1: 1.64% ; Q2: 3.43% ; Q3: 6.34% ; Q4 : 10.89%

© 2019 Atramont A et al. *JAMA Network Open*.

eTable 6. Characteristics of the Patients According to the 3-Year Mortality Risk After Hospital Discharge Among 3-Month Survivors

	**Total**		**Quintiles**									
			**1**		**2**		**3**		**4**		**5**	
	N	**%**	N	**%**	N	**%**	N	**%**	N	**%**	N	**%**
	101 207		20 309		20 175		20 241		20 241		20 241	
**Age (years)**												
18-34	9 452	**9.3**	8 748	**43.1**	598	**3.0**	84	**0.4**	21	**0.1**	1	**0.0**
35-44	8 400	**8.3**	3 355	**16.5**	3 971	**19.7**	838	**4.1**	201	**1.0**	35	**0.2**
45-54	14 143	**14.0**	2 384	**11.7**	4 325	**21.4**	4 894	**24.2**	1 846	**9.1**	694	**3.4**
55-64	21 841	**21.6**	3 280	**16.2**	2 743	**13.6**	7 420	**36.7**	5 195	**25.7**	3 203	**15.8**
65-69	12 168	**12.0**	1 509	**7.4**	1 821	**9.0**	2 923	**14.4**	3 447	**17.0**	2 468	**12.2**
70-74	10 297	**10.2**	670	**3.3**	2 229	**11.0**	1 497	**7.4**	3 237	**16.0**	2 664	**13.2**
75-79	10 874	**10.7**	363	**1.8**	2 532	**12.6**	1 261	**6.2**	3 333	**16.5**	3 385	**16.7**
80-84	8 838	**8.7**	.	.	1 757	**8.7**	806	**4.0**	2 409	**11.9**	3 866	**19.1**
85-89	4 106	**4.1**	.	.	199	**1.0**	488	**2.4**	509	**2.5**	2 910	**14.4**
90 and older	1 088	**1.1**	.	.	.	.	30	**0.1**	43	**0.2**	1 015	**5.0**
**Gender**												
Male	60 373	**59.7**	11 300	**55.6**	11 767	**58.3**	11 279	**55.7**	12 531	**61.9**	13 496	**66.7**
Female	40 834	**40.3**	9 009	**44.4**	8 408	**41.7**	8 962	**44.3**	7 710	**38.1**	6 745	**33.3**
**Comorbidities**			20 145	99	19 481	97	19 192	95	19 163	95	14 747	73
Heart failure	8 479	**8.4**	164	**0.8**	694	**3.4**	1 049	**5.2**	1 078	**5.3**	5 494	**27.1**
Cerebrovascular disease	4 656	**4.6**	200	**1.0**	669	**3.3**	745	**3.7**	1 180	**5.8**	1 862	**9.2**
Diabetes	21 252	**21.0**	1 433	**7.1**	3 829	**19.0**	3 321	**16.4**	5 559	**27.5**	7 110	**35.1**
Active cancer	8 391	**8.3**	21	**0.1**	195	**1.0**	728	**3.6**	1 418	**7.0**	6 029	**29.8**
Dementia (including Alzheimer’s disease)	1 644	**1.6**	5	**0.0**	26	**0.1**	100	**0.5**	228	**1.1**	1 285	**6.3**
Chronic respiratory diseases	18 753	**18.5**	649	**3.2**	2 031	**10.1**	2 326	**11.5**	5 029	**24.8**	8 718	**43.1**
End-stage renal disease	2 407	**2.4**	274	**1.3**	307	**1.5**	276	**1.4**	403	**2.0**	1 147	**5.7**
Liver diseases	4 750	**4.7**	147	**0.7**	538	**2.7**	695	**3.4**	1 269	**6.3**	2 101	**10.4**
**Cause of hospitalization**												
Cardiac surgery	21 908	**21.6**	8 600	**42.3**	9 582	**47.5**	2 721	**13.4**	804	**4.0**	201	**1.0**
Respiratory diseases	14 827	**14.7**	774	**3.8**	1 007	**5.0**	2 193	**10.8**	4 492	**22.2**	6 361	**31.4**
Cardiovascular diseases	7 986	**7.9**	399	**2.0**	675	**3.3**	1 858	**9.2**	2 355	**11.6**	2 699	**13.3**
Neurologic diseases	4 812	**4.8**	796	**3.9**	1 000	**5.0**	1 486	**7.3**	980	**4.8**	550	**2.7**

© 2019 Atramont A et al. *JAMA Network Open*.

Non-cardiac surgery	28 140	**27.8**	2 568	**12.6**	2 881	**14.3**	8 215	**40.6**	7 776	**38.4**	6 700	**33.1**
Poisonings	5 948	**5.9**	2 331	**11.5**	2 272	**11.3**	876	**4.3**	331	**1.6**	138	**0.7**
Hepatogastroenterology	3 375	3	187	1	318	**1.6**	634	**3.1**	1 079	**5.3**	1 157	**5.7**
Renal or metabolic diseases	4 022	**4.0**	379	**1.9**	385	**1.9**	975	**4.8**	1 194	**5.9**	1 089	**5.4**
Trauma and burn injuries	3 461	**3.4**	2 307	**11.4**	784	**3.9**	263	**1.3**	78	**0.4**	29	**0.1**
Organ transplant	1 651	**1.6**	766	**3.8**	528	**2.6**	229	**1.1**	110	**0.5**	18	0
Iinfectious diseases	1 691	**1.7**	114	**0.6**	158	**0.8**	372	**1.8**	470	**2.3**	577	**2.9**
Miscellaneous	3 386	3	1 088	5	585	**2.9**	419	**2.1**	572	**2.8**	722	**3.6**
**ICU procedures**												
Invasive mechanical ventilation	56 557	**55.9**	12 891	**63.5**	13 535	**67.1**	11 180	**55.2**	9 985	**49.3**	8 966	**44.3**
Non-invasive mechanical ventilation	26 716	**26.4**	2 248	**11.1**	4 196	**20.8**	4 193	**20.7**	6 771	**33.5**	9 308	**46.0**
Intravenous injection of dobutamine or dopamine	31 785	**31.4**	5 088	**25.1**	6 680	**33.1**	6 144	**30.4**	6 964	**34.4**	6 909	**34.1**
Intravenous infusion of filling product	16 034	**15.8**	2 496	**12.3**	2 508	**12.4**	3 540	**17.5**	3 970	**19.6**	3 520	**17.4**
Administration of blood products	5 576	**5.5**	1 184	**5.8**	1 317	**6.5**	996	**4.9**	1 071	**5.3**	1 008	**5.0**
Cardiopulmonary resuscitation with intubation	977	**1.0**	89	**0.4**	159	**0.8**	164	**0.8**	263	**1.3**	302	**1.5**
Renal replacement therapy	6 650	**6.6**	439	**2.2**	794	**3.9**	1 120	**5.5**	1 899	**9.4**	2 398	**11.8**
Emergency external electric shock	770	**0.8**	97	**0.5**	149	**0.7**	145	**0.7**	195	**1.0**	184	**0.9**
Intracranial pressure monitoring	1 706	**1.7**	738	**3.6**	514	**2.5**	332	**1.6**	91	**0.4**	31	**0.2**
Mechanical circulatory support	894	**0.9**	202	**1.0**	296	**1.5**	194	**1.0**	134	**0.7**	68	**0.3**

Q1:
6.36% ;
Q2:
12.31% ;
Q3:
20.54% ;
Q4 :
32.91%

© 2019 Atramont A et al. *JAMA Network Open*.

eTable 7. Factors Associated With Mortality in Logistic Regression Models With ICU Procedures Not Considered

	**In-hospital vital status**					
	n = 133 966					
	**Alive**		**Dead**			
	n = 108 539		n = 25 427			
	N	**%**	N	**%**	**OR**	95% CI
**Age (years)**						
18-34	9 582	**8.8**	540	**2.1**	**1.00**	Ref.
35-44	8 612	**7.9**	902	**3.5**	**1.95**	[1.74-2.18]
45-54	14 752	**13.6**	2 368	**9.3**	**2.94**	[2.66-3.25]
55-64	23 234	**21.4**	4 665	**18.3**	**3.76**	[3.41-4.13]
65-69	13 068	**12.0**	3 076	**12.1**	**4.58**	[4.14-5.05]
70-74	11 166	**10.3**	2 964	**11.7**	**5.32**	[4.82-5.88]
75-79	11 954	**11.0**	3 800	**14.9**	**6.36**	[5.77-7.02]
80-84	9 953	**9.2**	3 867	**15.2**	**7.66**	[6.94-8.45]
85-89	4 862	**4.5**	2 418	**9.5**	**8.88**	[8.01-9.86]
90 and older	1 356	**1.2**	827	**3.3**	**10.11**	[8.91-11.48]
**Gender**						
Male	64 851	**59.7**	15 445	**60.7**	**1.00**	Ref.
Female	43 688	**40.3**	9 982	**39.3**	**0.89**	[0.86-0.91]
**Comorbidities**						
Heart failure	9 770	**9.0**	3 890	**15.3**	**1.29**	[1.23-1.34]
Cerebrovascular disease	5 173	**4.8**	1 744	**6.9**	**1.06**	[1.00-1.12]
Diabetes	23 252	**21.4**	6 602	**26.0**	**1.02**	[0.98-1.05]
Active cancer	9 729	**9.0**	3 297	**13.0**	**1.26**	[1.21-1.32]
Dementia (including Alzheimer's disease)	1 987	**1.8**	1 042	**4.1**	**1.21**	[1.11-1.31]
Chronic respiratory diseases	20 821	**19.2**	6 094	**24.0**	**0.96**	[0.93-1.00]
End-stage renal disease	2 674	**2.5**	704	**2.8**	**1.04**	[0.95-1.13]

© 2019 Atramont A et al. *JAMA Network Open*.

Liver diseases	5 304	**4.9**	2 068	**8.1**	**1.78**	[1.68-1.88]
**Cause of hospitalization**						
Cardiac surgery	22 339	**20.6**	1 373	**5.4**	**1.00**	Ref.
Respiratory diseases	16 688	**15.4**	5 191	**20.4**	**5.22**	[4.89-5.57]
Cardiovascular diseases	8 969	**8.3**	4 475	**17.6**	**7.86**	[7.35-8.40]
Neurologic diseases	5 216	**4.8**	2 626	**10.3**	**10.24**	[9.51-11.03]
Non-cardiac surgery	30 068	**27.7**	6 186	**24.3**	**3.64**	[3.42-3.88]
Poisonings	6 056	**5.6**	261	**1.0**	**1.26**	[1.09-1.44]
Hepatogastroenterology	3 878	**3.6**	1 428	**5.6**	**6.04**	[5.55-6.57]
Renal or metabolic diseases	4 478	**4.1**	899	**3.5**	**3.33**	[3.04-3.65]
Trauma and burn injuries	3 561	**3.3**	826	**3.2**	**6.58**	[5.97-7.26]
Organ transplant	1 677	**1.5**	155	**0.6**	**1.85**	[1.54-2.21]
Infectious diseases	1 920	**1.8**	1 010	**4.0**	**9.22**	[8.37-10.16]
Miscellaneous	3 689	**3.4**	997	**3.9**	**6.80**	[6.20-7.47]

© 2019 Atramont A et al. *JAMA Network Open*.
